# Socio-economic and demographic factors associated with snacking behavior in a large sample of French adults

**DOI:** 10.1186/s12966-018-0655-7

**Published:** 2018-03-15

**Authors:** Wendy Si Hassen, Katia Castetbon, Sandrine Péneau, Christine Tichit, Anouar Nechba, Aurélie Lampuré, France Bellisle, Serge Hercberg, Caroline Méjean

**Affiliations:** 1 Centre de Recherche en Epidémiologie et Statistique, Equipe de Recherche en Epidémiologie Nutritionnelle (EREN), Inserm (U1153), Inra (U1125), Cnam, Université Paris 13, 74 rue Marcel Cachin, F-93017 Bobigny, France; 2 Université Libre de Bruxelles, Centre de Recherche en Epidémiologie, Biostatistique et Recherche Clinique, Ecole de Santé publique, Route de Lennik 808 – CP 598, 1070 Brussels, Belgium; 3 Institut National de la Recherche Agronomique (INRA) – UR 1303 Alimentation et Sciences Sociales ALISS, 65 boulevard de Brandebourg, F-94025 Ivry sur Seine, France; 4 Département de santé publique, Hôpital Avicenne, 125 rue de Stalingrad, F-93000 Bobigny, France; 5 MOISA, INRA, CIRAD, CIHEAM-IAMM, Montpellier SupAgro, Université de Montpellier, Montpellier, France

**Keywords:** Snack, Snacking, Socioeconomic position, Demographic factors, Nutritional content, Dietary behavior, Eating behavior

## Abstract

**Background:**

Few studies have specifically focused on demographic and socio-economic characteristics associated with snacking in adults, whereas their identification could be useful for defining effective public health measures. The aim of our study was to assess the associations of these factors with daily snacking behavior and its dietary quality.

**Methods:**

This cross-sectional study included 84,692 women and 23,491 men from the NutriNet-Santé cohort study. Occurrence of snacking, energy intake from snacks, snack nutrient, and energy densities were assessed using 24-h dietary records of weekdays at baseline. Associations between socio-economic and demographic factors (age, presence of children in the household, education, income, occupation), and snacking behavior were examined using multivariable logistic regression and analysis of covariance, stratified by sex and adjusted for total daily energy intake.

**Results:**

Older individuals were more likely to snack during the day in both sexes while individuals with primary education (OR = 0.79 (0.71;0.87) in women; OR = 0.71 (0.60;0.83) in men), female employees (OR = 0.94 (0.89;0.99), and self-employed women were less likely to snack during the day. Older individuals, in particular middle-aged subjects, had higher snack nutrient density, and lower energy intake and density from snacks compared with younger adults. Presence of a child in the household was associated with higher energy density, lower nutrient density (in women), and lower energy intake from snacks (in men), compared with those who lived without a child in household. In low income individuals and manual workers, snacks had lower nutrient density and higher energy content than in higher socioeconomic categories. At last, energy intake from daily snacking occasions was higher in women with low education level.

**Conclusions:**

Although snacking was less prevalent in low socioeconomic categories and young adults, their snacks had higher energy content and were of poorer nutrient density. Such findings provide useful information on mechanisms of social disparities in dietary behavior.

**Trial registration:**

This study was conducted according to the guidelines laid down in the Declaration of Helsinki. All procedures were approved by the Institutional Review Board of the French Institute for Health and Medical Research (IRB Inserm No0000388FWA00005831) and the French Data Protection Authority (Commission Nationale Informatique et Libertés No.908450 and No.909216). Clinical Trial no. NCT03335644

**Electronic supplementary material:**

The online version of this article (10.1186/s12966-018-0655-7) contains supplementary material, which is available to authorized users.

## Background

Snacking appears to be common in Western countries [1–3]. The effects of snacking and eating frequency on dietary quality, nutrient intake, and weight status are unclear [4–7]. In some studies, snacking or high eating frequency are associated with better dietary quality and higher intake of vitamins, potassium or magnesium [4, 8–11]. In contrast, one Scandinavian study showed that, when snacks contribute to most of the daily energy, the nutrient quality of diet is lower compared to the diet of individuals for which energy intake from snacks is lower [12]. Other studies have shown that snacking may represent a high percentage of daily energy intake (up to 30-35%) [5] and may contribute to a positive energy balance [7, 13, 14] that could consequently lead to weight gain [15, 16].

Identification of population subgroups with frequent and unhealthy snacking behavior may help public health policymakers to target the dietary behavior of populations in a more focused and efficient manner. Some studies have assessed demographic and socioeconomic differences for snacking [8, 12, 13, 17–20] but they have mostly focused on snacking frequency [8, 13, 18]. These studies found that age was inversely associated with snack frequency and snacking behaviors [8, 12, 18, 20]. Sex differences in snacking behavior in adults have been reported but are contradictory [8, 13, 17–19]. Results on the associations between the presence of a child in the household and snacking behavior remain scarce and unclear: one study found that having a child in the household was inversely associated with a “snacker pattern” in women [20] while another study found no significant association between snack frequency and having children [18]. Regarding socioeconomic position, previous works on education showed equivocal results [8, 12, 18, 19] while income seems to be positively associated with snacking frequency [8, 19]. Regarding occupation, male manual workers were more likely to have a diet in which snacks contribute to most of the daily energy, than non-manual workers [12].

However, little is known about relationships between the nutritional quality of snacks and socioeconomic and demographic profiles [18, 19]. The few studies that investigated nutritional quality of snacks among these subgroups focused only on energy content of snacks or main groups consumed during these occasions. The aims of our study were therefore to assess associations between demographic and socioeconomic factors and occurrence of snacking, on one hand, and nutritional quality of snacks (energy content and nutrient and energy densities), on the other hand, in a large sample of French adults.

## Methods

### Population and design

Subjects were participants in the NutriNet-Santé study, a large web-based prospective observational cohort launched in France in May 2009 and for which recruitment of participants is still ongoing. The study was implemented in the French general population targeting internet-using volunteers aged ≥18 years. The design, methods and rationale have been described previously [21]. Briefly, participants were included in the cohort once they had completed a baseline set of questionnaires assessing dietary intake, physical activity, socioeconomic, and health status. As part of their follow-up, the participants completed the same set of questionnaires every year. This study was conducted according to the guidelines laid down in the Declaration of Helsinki, and all procedures were approved by the Institutional Review Board of the French Institute for Health and Medical Research (IRB Inserm No. 0000388FWA00005831) and the French Data Protection Authority (Commission Nationale Informatique et Libertés No. 908450 and No. 909216). Electronic informed consent was obtained from all participants.

This cross-sectional study focused on participants included in the NutriNet-Santé cohort study between May 2009 and January 2015 living in the mainland France, who had completed at least two 24-h dietary records at baseline, and with no missing data on socioeconomic and demographic factors. For each individual, we used data at baseline, collected between 2009 and 2015, according to the date of inclusion of the participant. Only weekday 24 h records were used in our analysis.

### Data collection, data computation and statistical analysis

#### Demographic and socioeconomic data

Socioeconomic and demographic data were collected at baseline using a web-based self-completed questionnaire, using categories consistent with the French National Institute of Statistics definitions [22–24]. Age and presence of children in the household (Yes/No) were collected. Four categories of age were used: < 30 y, 31-45y, and 46-60y and >60y. The highest attained diploma defined the educational level [22] and four categories were used in the analysis: primary education, secondary education, undergraduate (corresponding to up to 3 years after high school diploma), and postgraduate (> 3 years after high school diploma). Occupation was coded into 6 classes: never employed (homemakers, students, and disabled), manual workers, employees, intermediate professions (e.g., technicians, skilled employees, teachers, nurses), self-employed (craftsman, shopkeeper, company manager, and farmer), and managerial staff. When subjects were unemployed or retired, the occupational category of their last job was recorded. Participants reported their monthly household income including salary, social benefits, family allowance, and rental income. The reported monthly household income was then divided by the number of household units (HU): 1 HU is attributed for the first adult in the household, 0.5 HU for other persons aged ≥14 years, and 0.3 HU for children < 14 years [25]. The following four categories of monthly income were used: < 1200€, 1200-1800€, 1800–2700€, and > 2700€ per HU plus a category for individuals who were unwilling to answer.

#### Assessment of dietary behaviors

At baseline, participants were invited to fill in 3 non-consecutive web-based 24-h dietary records, randomly assigned over a 2-week period (2 weekdays and 1 weekend day) [21, 26, 27]. The dietary record was completed via a validated interactive interface and designed for self-administration on the Internet [28]. The web-based dietary assessment method relies on an event-based approach, recording all foods and beverages (type and quantity) consumed at breakfast, lunch, dinner, and all other eating occasions, thus leading to 4 initial categories of eating occasions. They estimated portion sizes for each reported food and beverage according to standard measurements (e.g., home containers, grams indicated on the package) or using validated photographs [29]. Values for energy, macronutrients, and micronutrients were estimated using published nutrient databases and completed for recent market foods and recipes [30]. Energy-underreporting participants were identified by the method proposed by Black [31]. Briefly, basal metabolic rate (BMR) was estimated by Schofield eqs. [32] according to sex, age, weight and height collected at enrolment in the study. BMR was compared to energy intake taking into account the physical activity level. A physical activity level of 1.55 was used to identify energy-underreporting subjects [31]. They were consequently excluded for analysis.

Because of variable and unusual eating behaviors on the weekends, we focused our analysis on weekdays. We categorized the eating occasions according to their nutritional content and self-reported time. Eight categories of eating occasions were used: 3 main meals (breakfast, lunch, dinner) and 5 snacks (morning, midday, afternoon, evening and night snacks). Daily overall snacking was defined by the occurrence of at least one snacking occasion during the 24 h record. All snacking occasions occurring at different times during a 24 h record were then pooled to define the nutritional content of overall daily snacking. A binary variable (Yes/No) indicating whether the individual had snacked at least once during the day was computed.

#### Energy intake and energy density

Energy intake of one snacking occurrence was calculated by summing energy intake associated with all food items consumed during this eating occasion. Energy intake from daily snacking occasions was calculated by summing the energy intake of all snacking occasions. Energy density was defined as the ratio of total energy (in kcal) by the weight (in g) of daily snack*100 [33]. Low-calorie beverages (first decile of energy per 100 g) were excluded of the computation.

#### Nutrient density

To assess daily nutrient density of snacks, the NRF9.3_100 kcal_ index [34] was used. The NRF9.3 is a score based on nine beneficial nutrients (protein, fibre, vitamins A, C, and E) and minerals (magnesium, potassium, iron, and calcium), and three nutrients that should be limited (saturated fat, added sugars, and sodium). Daily values defined by the Food and Drug Administration [35] were used to score each eating occasion using the NRF9.3 algorithm. A high positive score reflects dietary intake that provides large amounts of beneficial nutrients.

**Algorithm**: NRF9.3_100 kcal_ [34] = ∑_i = 1-9_(Nutrient_i_/RDV_i_)*100 - ∑_i = 1-3_(Nutrient_i_/MDV_i_)*100.

RDV = recommended daily values; MDV: maximum daily values.

#### Statistical analyses

Comparisons of socioeconomic and demographic characteristics and dietary intakes at baseline between men and women were performed using Student’s t-tests and Chi-square tests, as appropriate.

Independent associations between socioeconomic and demographic factors and snacking behaviors were examined using multivariable logistic regression for occurrence of snacking (at least one snacking occasion), and analysis of covariance for quantitative variables (nutrient and energy densities, and energy intake from daily snacks). The highest educated group, the highest income group, the managerial staff group, the lowest age class group, and the absence of children in the household were used as references in logistic regressions. Analysis of covariance is used to compare response means among socioeconomic and demographic groups (categorical socioeconomic and demographic variables) adjusted for a quantitative variable (total daily energy intake as covariate), thought to influence the outcome (nutrient density, energy density and energy intake from snacks as dependent variables respectively). Collinearity between the three SEP indicators was investigated by examining the variance inflation factor. First, models assessing the associations between each demographic and socio-economic factor and each outcome (nutrient and energy densities and energy intake from daily snacks) were performed. Then, the demographic and socio-economic indicators were included all together in logistic and covariance models. All models were adjusted for total daily energy intake. As snacking occurrence and socioeconomic and demographic indicators have been associated with skipping meals [36–39], we considered the three binary variables (having breakfast (Yes/No), having lunch (Yes/No), and having dinner (Yes/No)) as confounding variables. These variables were therefore included in model of snacking occurrence. All analyses were performed separately in men and women since interactions were found. Data treatment and statistical analyses were performed using SAS (version 9.3; SAS Institute, Inc., Cary, NC, USA).

## Results

### Description of the sample

Among 144,746 individuals with available dietary data at baseline, we excluded individuals who were pregnant, those who did not provide at least two 24-h dietary records (*n* = 19,987; 13.8%), underreporting subjects (*n* = 15,785; 10.9%), and individuals with missing data for sociodemographic and economic variables (*n* = 791; 0.7%) - leaving 108,183 individuals for analysis **(**84,692 women and 23,491 men). For all factors considered, differences between men and women were found (Table 1). In women, percentages of younger respondents (18-30 years), those with undergraduate education, employees, and never-employed, those with the lowest income, and those living in household with at least one child were higher than in men; conversely, higher percentages of older adults (> 60 years), those with post-graduate education, managerial professions and self-employed persons, and those with the highest income level were observed in men compared with women (Table 1).

**Table 1:** Characteristics of participants in the NutriNet Santé Study included in the present study (*N* = 104,183; 23,491 men and 84,692 women)^a^

		Men (*N* = 23,491) % or mean (SD)	Women (*N* = 84,692) % or mean (SD)	*P*- value for chi-square or student t test analysis
Age class	≤30y	17.8	28.5	< 0.0001
	31-45	28.2	31.0	
	46-60	27.9	29.0	
	>60y	26.1	11.5	
Presence of children in the household	No child in the household	70.9	64.4	< 0.0001
	At least one child in the household	29.1	35.6	
Educational level	Postgraduate level	38.4	31.3	< 0.0001
	Undergraduate level	24.6	32.3	
	Secondary school	33.5	33.7	
	Primary school	3.5	2.7	
Household income per consumption unit	> 2700 euros	31.5	20.6	< 0.0001
	1800-2700 euros	25.2	22.2	
	1200-1800 euros	23.1	25.0	
	< 1200 euros	13.1	19.0	
	Unwilling to declare	7.1	13.2	
Occupation	Managerial staff	50.0	29.7	< 0.0001
	Self-employed	5.3	3.3	
	Intermediate profession	22.8	26.7	
	Employee	14.2	34.0	
	Manual worker	5.3	1.9	
	Never employed	2.4	4.4	

^a^Data used in the analysis were collected for each participant at inclusion in the cohort

### Snacking behaviors

Percentages of individuals having at least one snack and information regarding snack frequency are shown in Table 2. Higher occurrence of snacking during weekdays was observed in women compared to men (73.5% vs 65.4%, *p* < 0.0001). Among men and women who snacked, more than 80% had one or two daily snacks (Table 2). Means values regarding descriptive snacking characteristics of the sample are shown in Table 2 (energy intake, nutrient density, energy density and contribution to daily energy intake). Mean energy intake from daily snacks and mean nutrient density of snacks in women were lower than in men while mean energy density was higher (energy, mean (SD): 231 (247) kcal in women vs 296 (309) kcal in men; nutrient density, mean (SD): 104 (299) in women and 115 (275) in men; energy density (mean (SD): 232 (171) in women and 225 (170) in men; all *p*-values< 0.0001) (Table 2). In both sexes, among individuals who snack, snacking had an important contribution to total daily intake (approximately 12% in men and 13% in women (means)) (Table 2).

**Table 2:** Snacking characteristics of participants in the NutriNet Santé Study included in the present study (*N* = 104,183; 23,491 men and 84,692 women)^a^

	Men (*N* = 23,491) % or mean (SD)	Women (*N* = 84,692) % or mean (SD)	*P*- value for chi-square or student t test analysis
Having at least one snack	65.4	73.5	< 0.0001
Frequency of snack among individuals having at least one snack during the record			< 0.0001
1 snack per day	55.0	54.9	
2 snacks during the day	30.1	31.6	
3 snacks during the day	11.3	10.9	
4 snacks during the day	3.2	2.3	
5 snacks during the day	0.4	0.3	
Daily energy intake from snacking occasions (kcal)^b^	269.3 (309.0)	231 (246.6)	< 0.0001
Contribution of energy intake from snacking to total daily energy ^b,c^	11.8 (12.4)	12.8 (12.1)	< 0,0001
Daily mean nutrient density of snacks^b^	115.3 (274.8)	103.5 (299.1)	< 0.0001
Daily mean energy density (without low caloric beverages) of snacks (kcal/100 g)^b^	224.5 (170.2)	231.9 (170.8)	< 0.0001

^a^Data used in the analysis were collected for each participant at inclusion in the cohort

^b^Among individuals having at least one snack during the record

^c^Computed as (energy intake from daily snacks/total daily energy intake)*100

Median of energy intake from daily snacks of snacks in women were lower than in men while energy density and contribution to total daily intake were higher (energy intake, median (interquartile range): 162.0 (252.7) kcal in women vs 173.7 (300.2) kcal in men; energy density (median (interquartile range):170.8 (334.0) in women and 162.9 (338.2) in men; contribution to total daily intake median (interquartile range): 9.7 (14.1) in women and 8.2 (13.4) in men) (Additional file 1: Table S1).

#### Age

In both sexes, we observed bell-shape associations between age and occurrence of snacking, and between age and nutrient density of snacks (Tables 3 and 4). Middle-aged subjects, in particular those aged 31-45y and 46-60y were more likely to have at least one snacking occasion during weekdays compared to younger subjects (Table 3). Nutrient density of snacks of middle aged subjects was higher compared to younger adults (range according to sex: 103 to 122 in individuals aged 31-45y and 112 to 115 in individuals 46-60 y vs. 73 to 97 in participants aged 18-30y) (Table 4). In both sexes, energy density of snacks and energy intake from daily snacks decreased with age (range (youngest to oldest individuals); energy density 238 to 219 kcal/100 g in women (*p* < 0.0001) and 226 to 213 kcal/100 g in men (*p* = 0.004); energy intake from snacks: 266 to 220 kcal in women (*p* < 0.0001) and 341 to 257 kcal in men (*p* < 0.0001)) (Table 4).

**Table 3:** Associations^a^ between socioeconomic and demographic characteristics and snacking occurrence in women (N = 84,962) and men (*N* = 23,491)^b^

		Women			Men		
		Odds Ratio	Confidence Interval (CI 95%)	*P*-value	Odds Ratio	Confidence Interval (CI 95%)	*P*-value
Age class	≤30y	1		< 0.0001	1		0.0001
	31-45	1.24	1.18; 1.30		1.17	1.06; 1.29	
	46-60	1.35	1.29; 1.41		1.11	1.01; 1.22	
	>60y	1.14	1.08; 1.21		0.98	0.89; 1.08	
Presence of children in the household	No child at in the household	1		0.3	1		0.2
	At least one child in the household	1.02	0.98; 1.06		1.05	0.98; 1.13	
Educational level	Postgraduate level	1		< 0.0001	1		< 0.0001
	Undergraduate level	0.94	0.90; 0.98		0.97	0.90; 1.05	
	Secondary school	0.82	0.79; 0.87		0.87	0.80; 0.94	
	Primary school	0.79	0.71; 0.87		0.71	0.60; 0.83	
Household income per consumption unit	> 2700 euros	1		0.04	1		0.2
	1800-2700 euros	1.04	0.99; 1.09		0.97	0.90; 1.05	
	1200-1800 euros	1.03	0.98; 1.08		0.98	0.90; 1.06	
	< 1200 euros	0.98	0.92; 1.03		0.90	0.81; 1.00	
	Unwilling to declare	0.97	0.92; 1.03		0.89	0.79; 1.01	
Occupation class	Managerial staff	1		< 0.0001	1		0.02
	Self-employed	0.89	0.82; 0.98		0.93	0.82; 1.06	
	Intermediate profession	1.06	1.01; 1.11		1.11	1.03; 1.21	
	Employee	0.94	0.89; 0.99		1.06	0.96; 1.16	
	Manual worker	0.94	0.83; 1.06		1.14	0.99; 1.32	
	Never employed	0.97	0.89; 1.06		1.20	0.97; 1.48	

^a^Using multivariable logistic regression adjusted on total energy intake. The demographic and socio-economic factors were included simultaneously in the models

^b^Data used in the analysis were collected for each participant at inclusion in the cohort

**Table 4:** Associations^a^ between socioeconomic and demographic characteristics and daily nutrient density, energy intake and density of daily snacks in women (N _overall sample_ = 84,962; N_snacking_ = 62,209) and men (N_overall sample_ = 23,491; N_snacking_ = 15,359)

		Women									Men								
		Nutrient density^b^ of snacks			Energy density of snacks (kcal/100 g)			Energy intake from daily snacks (kcal)			Nutrient density^b^ of snacks			Energy density of snacks (kcal/100 g)			Energy intake from daily snacks (kcal)		
		Mean	SE	*p* value	Mean	SE	*P* value	Mean	SE	*P* value	Mean	SE	*p* value	Mean	SE	*P* value	Mean	SE	*P* value
Age class	≤30y	73.0	3.5	< 0.0001	237.5	2.1	< 0.0001	266.3	2.6	< 0.0001	96.6	6.6	< 0.0001	225.7	4.3	0.004	340.9	6.9	< 0.0001
	31-45	102.9	3.4		231.5	2.0		242.3	2.6		122.4	5.9		229.3	3.9		305.3	6.2	
	46-60	111.5	3.4		223.4	2.0		229.8	2.6		114.7	6.0		223.9	4.0		275.6	6.3	
	>60y	92.1	4.6		218.6	2.7		219.6	3.4		91.7	6.7		213	4.4		256.6	7.0	
Presence of child in the household	No child	99.9	2.9	0.0007	220.5	1.7	< 0.0001	241.7	2.2	0.05	102.3	4.8	0.1	217.9	3.1	0.005	308.0	5.0	< 0.0001
	At least one child	89.9	3.5		234.9	2.1		237.4	2.6		110.4	6.0		228	3.9		281.2	6.3	< 0.0001
Educational level	Postgraduate level	99.4	3.5	0.5	236.2	2.1	< 0.0001	229.3	2.6	<.0001	99.0	5.8	0.08	230.1	3.8	0.008	286	6.1	0.2
	Undergraduate level	97.0	3.1		231.7	1.9		232.2	2.3		95.8	5.6		231.4	3.7		292.5	5.9	
	Secondary school	95.2	2.8		225.8	1.7		238.7	2.1		99.4	4.7		219.9	3.0		300.4	4.9	
	Primary school	88	7.7		217.3	4.6		257.9	5.8		131.2	12.9		210.5	8.5		299.5	13.5	
Household income per consumption unit	> 2700 euros	111.4	3.97	< 0.0001	231.8	2.4	< 0.0001	220.1	3.0	< 0.0001	130.0	6.6	< 0.0001	227.1	4.3	0.1	271.9	6.9	< 0.0001
	1800-2700 euros	101.6	3.75		232.0	2.2		233.3	2.8		107.6	6.3		220.3	4.1		286.2	6.6	
	1200-1800 euros	91.0	3.5		229.9	2.1		240.3	2.6		106.0	6.0		226.9	3.9		284.3	6.3	
	< 1200 euros	85.2	3.66		219.9	2.2		259.5	2.8		96.3	6.9		216.7	4.5		325.2	7.3	
	Unwilling to declare	85.3	4.13		225.0	2.5		244.6	3.1		91.8	9.1		223.9	5.9		305.4	9.5	
Occupation class	Managerial staff	110.5	3.28	< 0.0001	230.7	2.0	0.2	220.3	2.5	< 0.0001	130.1	5.3	0.0001	222.9	3.5	0.9	261.1	5.5	< 0.0001
	Self-employed	101.1	6.97		225.3	4.2		232.2	5.2		121.4	10.4		227.3	6.8		291.7	10.8	
	Intermediate profession	94.6	3.11		230.2	1.9		235.2	2.3		106.4	5.9		219.8	3.8		274.2	6.1	
	Employee	92.6	2.87		225.9	1.7		240.2	2.2		100.5	6.8		221.4	4.4		295	7.1	
	Manual worker	90.3	8.99		224.4	5.3		252.7	6.8		91.7	10.1		222.6	6.5		316.4	10.5	
	Never employed	80.2	6.51		230	3.8		256.7	4.9		88.1	14.9		223.9	9.6		329.1	15.7	

^a^Using analysis of covariance adjusted on total energy intake. The demographic and socio-economic factors were included simultaneously in the models

^b^Calculated using the NRF9.3_100kcal_ score

^c^Data used in the analysis were collected for each participant at inclusion in the cohort

#### Presence of children in the household

In both sexes, no significant association between the presence of children in the household and snacking occurrence during the day was found (Table 3). In women only, daily nutrient density of snacks was lower in individuals living in a household with children compared to those living with no children (mean (SE): 89.9 (3.5) vs 99.9 (2.9) *p* = 0.0007) (Table 4). In both sexes, daily energy density of snacks was higher when at least one child was present in the household (in women (mean (SE)): 234.9 (2.1) kcal/100 g vs 220.5 (1.7) kcal/100 g) (Table 4). In men only, energy intake from daily snacking was lower in men who lived with a child than in those living with no children (Table 4).

#### Socioeconomic position (SEP)

Overall, variance inflation factor of each SEP indicator was between 1.01 and 1.64, showing that SEP indicators were not collinear.

#### Education

Low education was associated with lower occurrence of snacking during weekdays in both sexes (Table 3). In both sexes, energy density of snacks was lower in low education groups compared to the postgraduate level (− 20 kcal/100 g between individuals with primary school level and those with postgraduate level, *p* < 0.0001) (Table 4). In women only, energy intake from daily snacks decreased with education level (+ 29 kcal in snacks of women with primary school level compared to those with postgraduate level) (Table 4).

#### Occupation

In both sexes, individuals with intermediate professions were more likely to snack during weekdays than managerial staff participants (Table 3). In women, employees and self-employed individuals were less likely to snack than women with managerial positions (Table 3). Daily nutrient density of snacks was lower in manual workers and employees compared to those belonging to the managerial category (− 20 to − 40 according to sex) (Table 4).

Energy intake from daily snacks of manual workers and employees was higher compared to individuals belonging to the managerial category (+ 20 to + 30 kcal in women and + 34 to + 55 kcal in men) (Table 4). Energy intakes from daily snacks are minimal in individuals with managerial professions (mean (SE): 220.3 (2.5) in women and 261.1 (5.5) in men) and maximal for manual workers (mean (SE): 252.7 (6.8) in women and 316.4 (10.5 in men) among professionally active individuals.

#### Income

No significant difference in snacking occurrence during weekdays was found according to income levels (Table 3). Daily nutrient density of snacks was lower and energy intakes of daily snacks was higher in low income individuals compared to those with higher income (Table 4). For instance, we observed that daily snacks of individuals with the lowest income had a mean additional energy intake of 40 kcal in women and 55 kcal in men compared to snacks of those with the highest income. In women only, energy density increased with income (Table 3).

## Discussion

This study presents an original assessment of differences in both occurrence and nutritional quality of snacking according to socioeconomic and demographic factors in adults. Compared to younger adults, older individuals were more likely to snack and the nutritional quality of their snack was higher. Presence of children in the household was associated with higher energy density of snacks in both sexes and lower nutrient density of snacks in women. Snacking behavior was less prevalent in low educated individuals and varied according to occupation levels. However, compared with those from the highest socioeconomic groups, nutritional quality of snacks in low socioeconomic groups was lower (lower nutrient density and higher energy intake from snacks).

### Age

In our study, middle aged individuals were more likely to snack during the day. A previous work has shown that age was inversely associated with snack frequency while another study showed that the higher snack frequency was found among individuals aged 40-59y [8]. Individuals aged 30y and more are more likely to be in the workforce compared with younger adults or individuals aged 60y or more and the workplace has been associated with snacking [40]. Indeed, self-control imposed by work, emotional affects, daily hassles, and working hours along with social context of eating and availability of foods at work could lead working adults to snack during the day, even in the absence of physiological hunger [41–44]. Higher nutritional quality of snacks of middle-aged individuals are linked to higher contribution of fruits, hot beverages, and lower contribution of fatty and sweet products to the energy intake of daily snacks in these age classes (data not shown). Nutrient density of men aged 60 y or more was lower than that of the youngest male individuals because alcohol contribution to snack energy is higher in men aged 60y and more (data not shown).

### Presence of children in the household

Presence of at least one child in the household was not significantly associated with snacking occurrence while it was associated with lower nutritional quality of snacks, particularly in women. Along with increased availability at home [45–49], adults may consume products usually eaten by children during snacks such as biscuits, bread, pastries, cereals or chocolate [50], contributing to the lower nutritional quality. In further analyses (Additional file 2: Table S2), we observed in women that having children in the household was associated with a lower contribution of fruits and with a higher contribution of sugary products and fatty sweet foods to total energy intake from snack. In addition, in our cohort, women who had at least one child were more likely to have a strong liking for the fat-and-sweet sensation compared with those without children [51].

### Socioeconomic position

In contrast with previous studies [12, 18], our findings showed that individuals belonging to low SEP (education and occupation) were less likely to snack. Such differences could be explained by heterogeneity in study design. Indeed, our study did not investigate snacking frequency but occurrence of snacking and the definition of snacking was not based on the participant’s self-declaration of “snacks” in contrast to previous works. In addition, contribution of snacking to total energy intake seems to be considerably higher in countries such as the USA, the Netherlands or Scandinavian countries (up to 35% of daily energy) [5, 52] than in France (around 13% in our sample, 18% in another French study [1]). Snacking behaviors are thus more anchored in those countries than in France, where the “three main meals” pattern remains important [53]. Consequently, in these countries, snacking behaviors are more likely to reflect socioeconomic disparities in overall diet whereas difference regarding snacking occurrence in our study may rather show behavioral differences among social groups. In line with previous studies showing that low socioeconomic status is associated with poorer nutrient intakes and food intakes [54–56], low SEP individuals had overall lower nutritional quality of snacks. As snacks of low SEP individuals had higher energy content than those of high SEP, snacking of low SEP might lead particularly to excess daily energy intake and consequently, to positive energy balance which is a risk factor of weight gain [15]. However, the snacks of high SEP individuals had higher energy density compared with those of the lower SEP categories. These data suggest that high SEP individuals consume more energy dense products but in a small amount.

Employees and self-employed individuals were less likely to snack in our study compared with managerial staff individuals. Managerial individuals may have extensive discretion in organising their work, leaving them more occasions to snack. In addition, they are more likely to have more conviviality eating occasions (meeting, cocktail and farewell parties, conference break…) in the professional environment [57, 58].

An important body of literature has concluded that a high education level is associated with healthy dietary patterns including a greater consumption of fruits, vegetables, and whole-grain foods [55, 56, 59]. However, our results regarding nutritional quality of snacking are more mixed. Individuals with lower education levels had snacks with lower energy density compared to those of higher education levels. In further analyses (data not shown), we observed that fatty sweet products had a lower contribution to energy intake from daily snacks in individuals with lower education levels than in highly educated persons. Our web-based design and our method of assessment of dietary behaviors may have reduced desirability bias in high educated individuals, leading them to report more accurately their consumption of energy-dense products, even if these individuals are aware of its health impact [60, 61].

We found that lower income was associated with lower nutrient density of snacks. The higher cost of nutrient-dense products may lead individuals with low income to choose foods with lower nutritional value and may explain the differences in nutritional quality of snack according to income classes [62]. In addition, the perception of the association between nutrition and health can vary according to income level. Compared to low income individuals, high income individuals are more exposed to nutrition knowledge and therefore more likely to make food choices that take health consideration into account [63–65].

As snacking seems to be a common behavior that contributes significantly to daily energy intake (12-13% of total energy intake among individuals who snack), it could impact negatively diet quality and health. Some individuals seem more likely to snack during the day such as older high educated or high occupational individuals. The present study showed that lower nutritional quality of snacks (particularly, nutrient density and energy intake) is associated with presence of child in women only, younger age and low socioeconomic position. This suggests less healthy behaviors in these specific subgroups of the French population. When focusing on snacking, policy makers and practitioners should integrate the importance of these determinants in their recommendations. Since snacking is prevalent and could help to avoid overconsumption at the subsequent meal [66], it could be interesting to promote healthy snacking (fruits, vegetables, low fat and low sugar dairy products…), particularly among individuals of lower socioeconomic position. For instance, implementation of actions through professional networks or short education programs should be considered. In addition, actions focusing on food reformulation and changes in food supply could help reducing socioeconomic disparities in nutritional quality of snacks. Among possible changes, easy to read front-of-pack labelling based on nutrient profiling would allow consumers to compare snack products and might encourage food industries to improve the nutritional quality of their products.

#### Strengths and limitations

Since the NutriNet-Santé cohort includes volunteers, more subjects were women, belonged to high education group and had a healthier lifestyle [67, 68], and were probably more interested in nutrition than the general population. Thus, caution is needed when interpreting and generalizing results. A web-based design may affect internal validity by inducing misreporting. However, studies investigating the validity of our web-based, self-reported dietary record tool against biomarkers showed that our tool performs well at estimating several nutrient and food intakes [26, 27]. The issue of accuracy of web-based self-reported data also arises for repeated 24-h dietary records compared to interviews by trained dietitians but previous work showed high agreement between the two methods in the case of our study [28].

Contrary to previous work, the definition of snacking was not based on food items consumed or the participant’s self-definition of snacking. The fact that the volunteers did not declare snacking occasions as such (but only eating occasions in general) may have reduced the desirability bias, since snacking is often viewed as unhealthy behavior. Regarding socioeconomic data, a previous study showed that the quality of information provided by the web-based socio-demographic questionnaires used in the study was equal to or better than that of the paper-based questionnaire [23]. In addition, the large size of our sample may have been a constraint since significant results were found even when the difference between groups was small. However, the sample size and the diversity of collected data about demographic and socioeconomic factors enabled a highly accurate estimate and adjustment for several confounders. A major strength of our study lies in its reliance on several demographic factors and the use of the three major indicators of SEP. The use of demographic and socioeconomic indicators simultaneously allowed specific relationships to be highlighted which provide useful information to a better understanding of the mechanisms leading to social inequalities in health. The different parameters used to assess the nutritional quality of snack (energy intake, nutrient and energy densities) constitute an original contribution. Since the NRF9.3_100kcal_ has been validated and can be applied to individual food, meals, menus, and even the daily diet, this nutrient index enabled us to assess the nutrient density of snacking occasions [34, 69]. The strength of this score lies in the fact that it includes both positive and negative components.

## Conclusions

Our study highlights that snacking practices vary depending on demographic and socioeconomic factors. Age, education, and occupation appear to be predictors of occurrence of snacking. Having children in the household and low socioeconomic status seem to be associated with less healthy snacking behaviors. Better knowledge of the social disparities in snacking could help implementing public health policies, particularly in at risk populations. Further work assessing the mediating effect of snacking behaviors in the associations between socioeconomic position and weight and health status would be useful.

## Additional files

Additional file 1: **Table S1.** Medians and interquartile ranges of several snacking characteristics of participants in the NutriNet Santé Study included in the present study^a^. (DOCX 15 kb)

Additional file 2: **Table S2.** Associations^a^ between presence of child in the household and contribution^b^ of major foods groups to energy intake of daily snacks in women (N_overall sample_ = 84,962; N_snacking_ = 62,209) and men (N_overall sample_ = 23,491; N_snacking_ = 15,359)^b^. (DOCX 22 kb)

## Abbreviations

MDV: Maximum daily values
NRF9.3100 kcal: Nutrient-rich food index 9.3 per 100 kcal
RDV: Recommended daily values
SEP: Socioeconomic position
