# Supplementary material for: Socio-economic and demographic factors associated with snacking behavior in a large sample of French adults
Source: Int J Behav Nutr Phys Act. 2018 Mar 15;15:25. doi: 10.1186/s12966-018-0655-7 (PMC5856366; doi:10.1186/s12966-018-0655-7)
Supplement: Supplementary file 1 — Table S1. Medians and interquartile ranges of several snacking characteristics of participants in the NutriNet Santé Study included in the present studya. (DOCX 15 kb) [file 12966_2018_655_MOESM1_ESM.docx]

Additional file1 Table S1: Medians and interquartile ranges of several snacking characteristics of participants in the NutriNet Santé Study included in the present study^a^

|  | **Men** | **Women** |
| --- | --- | --- |
|  | **Median (interquartile range )** | **Median (interquartile range )** |
| **Daily energy intake from snacking occasions (kcal)^b^** | 173.7 (300.2) | 162.0 (252.7) |
| **Contribution of energy intake from snacking to total daily energy ^b,c^** | 8.2 (13.4) | 9.7 (14.1) |
| **Daily nutrient density of snacks^b^** | 22.2 (66.6) | 22.4 (64.7) |
| **Daily energy density (without low caloric beverages) of snacks (kcal/100 g)^b^** | 162.9 (338.2) | 170.8 (334.0) |
| a) Data used in the analysis were collected for each participant at inclusion in the cohort | | |
| b) Among individuals having at least one snack during the record | | |
| c) Computed as (energy intake from daily snacks/total daily energy intake)*100 | | |
