# Supplementary material for: Socio-economic and demographic factors associated with snacking behavior in a large sample of French adults
Source: Int J Behav Nutr Phys Act. 2018 Mar 15;15:25. doi: 10.1186/s12966-018-0655-7 (PMC5856366; doi:10.1186/s12966-018-0655-7)
Supplement: Supplementary file 2 — Table S2. Associationsa between presence of child in the household and contributionb of major foods groups to energy intake of daily snacks in women (Noverall sample = 84,962; Nsnacking = 62,209) and men (Noverall sample = 23,491; Nsnacking = 15,359)b. (DOCX 22 kb) [file 12966_2018_655_MOESM2_ESM.docx]

Additional file 2: Table S2: Associations^a^ between presence of child in the household and contribution^b^ of major foods groups to energy intake of daily snacks in women (N_overall sample_ =84,962; N_snacking_=62,209) and men (N_overall sample_=23,491; N_snacking_=15,359). ^b^

|  |  | **Fruits** | **P value** | **Bread** | **P value** | **Fatty sweet products** | **P value** | **Sweet products** | **P value** | **Sweet non alcoholic beverages** | **P value** | **Hot beverages** | **P value** | **Juices (fruit and vegetable)** | **P value** | **Alcoholic beverages** | **P value** | **Milk and milk substitues** | **P value** |
| --- | --- | --- | --- | --- | --- | --- | --- | --- | --- | --- | --- | --- | --- | --- | --- | --- | --- | --- | --- |
|  |  | **Mean percentage (SE)** |  | **Mean percentage (SE)** |  | **Mean percentage (SE)** |  | **Mean percentage (SE)** |  | **Mean percentage (SE)** |  | **Mean percentage (SE)** |  | **Mean percentage (SE)** |  | **Mean percentage (SE)** |  | **Mean percentage (SE)** |  |
| **Women** | No child | 14.4 (0.3) | <0.0001 | 5.6 (0.2) | <0.0001 | 31.3 (0.4) | <0.0001 | 7.6 (0.2) | 0.009 | 4.0 (0.2) | 0.0006 | 10.1 (0.3) | 0.4 | 3.6 (0.1) | <0.0001 | 2.1 (0.1) | <0.0001 | 4.2 (0.1) | 0.3 |
|  | At least one child | 12.6 (0.3) |  | 6.5 (0.2) |  | 35.2 (0.5) |  | 8.2 (0.3) |  | 3.4 (0.2) |  | 9.8 (0.3) |  | 3.0 (0.2) |  | 1.0 (0.1) |  | 4.4 (0.2) |  |
| **Men** | No child | 12.4 (0.5) | 0.4 | 5.5 (0.3) | 0.2 | 27.9 (0.7) | 0.2 | 9.2 (0.4) | 0.6 | 4.8 (0.3) | 0.3 | 11.1 (0.5) | 0.2 | 3.9 (0.3) | 0.2 | 5.9 (0.4) | 0.005 | 3.5 (0.2) | 0.2 |
|  | At least one child | 11.9 (0.6) |  | 5.9 (0.3) |  | 29.0 (0.9) |  | 9.5 (0.5) |  | 4.5 (0.4) |  | 11.9 (0.7) |  | 3.5 (0.4) |  | 4.8 (0.4) |  | 3.2 (0.3) |  |
| 1. Using analysis of covariance adjusted on total daily energy intake. The demographic and socio-economic factors were included simultaneously in the models. 2. Data used in the analysis were collected for each participant at inclusion in the cohort | | | | | | | | | | | | | | | | | | | |
